# Transmission of Chikungunya Virus in an Urban Slum, Brazil 

**DOI:** 10.3201/eid2607.190846

**Published:** 2020-07

**Authors:** Rosângela O. Anjos, Vánio André Mugabe, Patrícia S.S. Moreira, Caroline X. Carvalho, Moyra M. Portilho, Ricardo Khouri, Gielson A. Sacramento, Nivison R.R. Nery, Mitermayer G. Reis, Uriel D. Kitron, Albert I. Ko, Federico Costa, Guilherme S. Ribeiro

**Affiliations:** Fundação Oswaldo Cruz, Salvador, Brazil (R.O. Anjos, P.S.S. Moreira, C.X. Carvalho, M.M. Portilho, R. Khouri, G.A. Sacramento, N.R.R. Nery Jr., M.G. Reis, U.D. Kitron, A.I. Ko, F. Costa, G.S. Ribeiro);; Universidade Licungo, Quelimane, Mozambique (V.A. Mugabe);; Universidade Federal da Bahia, Salvador (V.A. Mugabe, R. Khouri, N.R.R. Nery Jr., M.G. Reis, F. Costa, G.S. Ribeiro);; Yale University, New Haven, Connecticut, USA (M.G. Reis, A.I. Ko, F. Costa);; Emory University, Atlanta, Georgia, USA (U.D. Kitron);; University of Liverpool, Liverpool, UK (F. Costa);; Lancaster University, Lancaster, UK (F. Costa)

**Keywords:** Arboviral infections, asymptomatic infection, Brazil, chikungunya virus, cross-sectional study, epidemics, epidemiology, herd immunity, outbreaks, seroprevalence, urban, vector-borne infections, viruses

## Abstract

After a chikungunya outbreak in Salvador, Brazil, we performed a cross-sectional, community-based study of 1,776 inhabitants to determine chikungunya virus (CHIKV) seroprevalence, identify factors associated with exposure, and estimate the symptomatic infection rate. From November 2016 through February 2017, we collected sociodemographic and clinical data by interview and tested serum samples for CHIKV IgG. CHIKV seroprevalence was 11.8% (95% CI 9.8%–13.7%), and 15.3% of seropositive persons reported an episode of fever and arthralgia. Infections were independently and positively associated with residences served by unpaved streets, a presumptive clinical diagnosis of chikungunya, and recall of an episode of fever with arthralgia in 2015–2016. Our findings indicate that the chikungunya outbreak in Salvador may not have conferred sufficient herd immunity to preclude epidemics in the near future. The unusually low frequency of symptomatic disease points to a need for further longitudinal studies to better investigate these findings.

In the 21st century, chikungunya virus (CHIKV) has emerged as a mosquitoborne disease of global relevance, causing large epidemics because of its widespread dissemination in tropical and subtropical areas (*1*). Infected persons usually develop an acute febrile illness associated with joint pains, myalgia, headache, and other signs and symptoms that can lead to misdiagnosis with other arboviral illnesses, such as dengue virus (DENV) and Zika virus (ZIKV) infections. Noteworthy with chikungunya, the arthralgia is often severely debilitating and may last for months to years (*1*,*2*). 

After the introduction of CHIKV into the Caribbean region in 2013, the virus spread rapidly, causing large outbreaks (*3*,*4*). In certain Caribbean islands, such as Puerto Rico and the US Virgin Islands, the rate at which CHIKV infection was symptomatic was estimated at >70% (*5*,*6*). However, it remains unclear whether the attack rates in outbreaks in large population centers in the Americas created sufficiently high levels of herd immunity to preclude subsequent epidemics. Furthermore, 2 CHIKV strains were introduced and are cocirculating in the Americas; it is unclear whether the proportions of symptomatic infections differ on the basis of strain type, population, or region. 

In Brazil, CHIKV was first detected in September 2014, almost simultaneously in the cities of Oiapoque, in the northern state of Amapá, where the Asian genotype was implicated (*7*), and in Feira de Santana, in the northeast state of Bahia, where the East/Central/South African (ECSA) genotype was detected (*7*,*8*). The virus spread rapidly throughout the country, reaching all states by 2015 (*9*), and peaked in 2016, when ≈280,000 probable cases were recorded (*10*). The northeast region was the most affected by CHIKV (*9*,*10*,*11*); this same area was also the most affected by ZIKV in 2015–2016 (*12*,*13*). 

In Salvador (population 2.9 million [*14*]), the capital of Bahia state, which is located ≈100 km from Feira de Santana, we retrospectively identified that CHIKV had been circulating since September 2014 (*15*), but outbreaks first occurred between June and November 2015 (*12*,*15*). In this study, we used the prevalence of CHIKV IgG as an indicator of all (i.e., symptomatic and asymptomatic) previous infection in a slum community of Salvador and assessed the proportion of cases in which these infections were symptomatic. In addition, we investigated factors potentially associated with prior CHIKV infection. 

## Methods 

### Study Site and Participant Selection

We performed a cross-sectional study in Pau da Lima, a poor community in Salvador characterized by high population density and substandard sanitation infrastructure (*16*,*17*). Since 2003, this community has been the site for several studies aiming to determine the epidemiology and the transmission dynamics of leptospirosis (*17*–*19*), dengue, and other arboviral diseases (*15*,*16*,*20*,*21*), as well as the burden of chronic noncommunicable diseases on the community and its residents (*22*,*23*). Detailed information about the sociodemographics of the Pau da Lima community, environment, and urban infrastructure has been previously described in these studies.

We surveyed the residents of 3 contiguous valleys in Pau da Lima from November 2016 through February 2017. During the enrollment process, we visited all households in the study site and invited all residents ≥5 years of age who slept ≥3 nights per week in the house to participate. 

### Data Collection

We used a standardized questionnaire during household visits to obtain data on participant demographic and socioeconomic conditions. Data collected were age, sex, self-reported skin color, education level, occupation or work, household per capita income, material of housing walls (wood or other material that is not brickwork, plastered or not plastered), quality of streets accessing house (paved or unpaved), and number of residents per household (Appendix). We also collected self-reported data on prior presumptive clinical diagnosis of DENV, ZIKV, and CHIKV infection and on history of fever, arthralgia, myalgia, rash, and pruritus, at any time after January 2015. This information covered health effects from the period immediately before and after the peak of CHIKV transmission in Salvador, which occurred during June–November 2015 (*12*,*15*). We recorded the duration of arthralgia among participants who reported this symptom. We conducted the interviews on computer tablets and used Research Electronic Data Capture software (REDCap; https://projectredcap.org/software/) to store the data (*24*)*.*

### Serologic Evaluation

During the household visits, we collected 10 mL of blood from each participant and transported the samples on the same day, stored at 2°C–8°C, to our laboratory at the Instituto Gonçalo Moniz, Fundação Oswaldo Cruz, in Salvador. We centrifuged the samples to obtain serum, which we aliquoted and stored at −20°C until evaluation. We tested serum samples by using the IgG ELISA technique (Euroimmun, https://www.euroimmun.com) to detect specific CHIKV IgG. 

For samples showing positive results for IgG, we then tested with a CHIKV IgM ELISA (InBios, https://inbios.com); we used the presence of IgM as a proxy for a more recent CHIKV infection than if there were no IgM. We interpreted both the CHIKV IgG and IgM ELISA results according to manufacturer instructions: CHIKV IgG absorbance/calibrator levels were negative at <0.8, indeterminate at ≥0.8 to <1.1, and positive at ≥1.1; CHIKV IgM absorbance/calibrator levels were negative at <0.9, indeterminate at ≥0.9 to <1.1, and positive at ≥1.1. We retested samples indicating indeterminant results on the initial test and considered the results obtained final. 

To confirm the accuracy of results from the IgG ELISA, we performed a blind plaque-reduction neutralization test (PRNT) of a stratified random sample of 60 serum samples (30 positive and 30 negative from the CHIKV IgG ELISA) for CHIKV to determine ≥90% reductions in plaque counts (PRNT_90_), as described elsewhere (*25*). To investigate whether cryoglobulinemia could have reduced the sensitivity of the IgG ELISA, we retested 100 samples, randomly selected from those that had been IgG negative, using a prewarmed (2 h at 37°C) and centrifuge protocol (*26*)*.*

### Data Analysis

We used absolute and relative frequencies or medians and interquartile ranges (IQR) to characterize the sociodemographics and also reported presumptive diagnoses and history of symptoms of study participants. We used χ^2^ or Wilcoxon rank-sum tests to compare the sex and age distribution for those who did agree to be enrolled in the study with the distribution for those who did not. We used a 2-tailed p value of <0.05 to define statistically significant differences. 

We calculated the prevalence of CHIKV IgG overall and according to participants’ characteristics and categorized continuous variables so we could estimate CHIKV seroprevalence by groups. We stratified age into ranges of 5–14 years, 15–39 years, and ≥40 years to account for the disproportionately young average age of the sample; we characterized education level as illiterate for participants who had never studied and literate for participants who had studied >1 year. We determined those living in poverty using the World Bank’s criteria for poverty in upper-middle-income countries of <$5.50/day (US dollars) per capita household income (*27*)*.* We obtained 95% CIs for the prevalence measures, adjusting them for the design effect of sampling households as clusters. 

We used bivariate and multivariate Poisson regression models with robust variance and adjustment for design effect to verify associations between previous CHIKV infection and the sociodemographic and clinical characteristics of participants. We calculated prevalence ratios with 95% CIs and included all variables that had bivariate analyses with a p value <0.20 in the multivariate analyses. We then used a backward selection method to build 2 final multivariate models, retaining variables with a p value <0.05. The first model included only sociodemographic variables to investigate their role in CHIKV infection, whereas the second model included only clinical characteristics to address their capacity to predict a positive serologic result. 

Among the participants with a positive CHIKV IgG ELISA, we estimated the frequencies of symptomatic CHIKV infection by calculating the proportion of those who reported fever simultaneously accompanied by arthralgia after January 2015, likely recent CHIKV infection by calculating the proportion of those with a positive IgM test result, and presumptive clinical suspicion of chikungunya by calculating the proportion of those who reported having received that diagnosis. Wilcoxon rank-sum test was used to compare the median duration of arthralgia between those reporting arthralgia accompanied by fever and those reporting only arthralgia. Poisson regression models with robust variance, adjusted for design effect, were used to compare sociodemographic and clinical characteristics between participants with symptomatic CHIKV infections and those with asymptomatic infections and between participants with likely recent and those with likely nonrecent CHIKV infections. We set a two-tailed p value <0.05 to define statistically significant differences. We performed data analysis using Stata version 14 software (StataCorp, https://www.stata.com) (*28*). 

### Ethics Considerations

This study was approved by the Research Ethics Committee of Gonçalo Moniz Institute, Oswaldo Cruz Foundation (CAAE n° 55904616.4.0000.0040). Before any study procedure, all participants ≥18 years of age signed an informed consent form; those <18 years of age who were able to read signed an informed assent, with their parents providing a signed consent. 

## Results 

### Participants Characteristics

Among the 2,651 eligible residents in the study site, 1,776 (67.0%) agreed to participate in this study. Those who consented were younger than those who refused (median age 26 years [IQR 16–40] vs. 35 years [IQR 21–46]; p <0.01). Of those who consented, a greater proportion were female (57.0%) than those who did not consent (52.0%; p<0.01). Most participants had a nonwhite (black or mixed) skin color (93.8%), lived in a household with a per capita income <$5.50/day (US dollars) (80.8%), and had not completed elementary school education (59.0%) or were illiterate (4.3%). 

### Prevalence of Previous CHIKV Infection and Associated Factors

Among the 1,772 (99.8%) participants from whom we collected and tested a blood sample, 209 (11.8%, 95% CI 9.8%–13.7%) had had a previous CHIKV infection, as determined by the detection of CHIKV IgG. Of the 30 random IgG ELISA positive samples tested by CHIKV PRNT_90_, 27 (90%) were positive; of the 30 random IgG-negative samples, all were also negative in PRNT_90_ (agreement 95%; kappa 90%). Of the 100 IgG-negative samples that we retested to evaluate whether cryoglobulinemia had reduced ELISA sensitivity, 2 (2%) returned positive results, but these results had low absorbance/calibrator levels (1.11 and 1.15) compared with those observed for the 209 positive samples (median 3.53, IQR 3.11–3.82). 

In bivariate analyses, prevalence of previous CHIKV infection did not differ by sex, skin color, poverty level, or number of residents per household (Table 1). However, we found a statistically significant association with other indicators of socioeconomic status, residing on unpaved streets and living in houses whose walls were unplastered or were made of wood or other materials; in addition, we found a nonsignificant trend of greater prevalence among participants who were older, illiterate, or reported not working (Table 1). Furthermore, the prevalence of previous CHIKV infection was statistically greater for participants who had received a presumptive clinical diagnosis of an infection by any of 3 cocirculating arboviruses—CHIKV, DENV, or ZIKV—and for those who reported having symptoms compatible with an arboviral infection—fever with arthralgia, myalgia, rash, or pruritus—after January 2015, when CHIKV emerged in Salvador (Table 1).

**Table 1:** Prevalence of previous chikungunya virus infection, determined by detection of IgG, by demographic and clinical characteristics, Salvador, Brazil, November 2016–February 2017

Characteristic	No. participants	No. positive (prevalence, %)	p value
Sociodemographic			
Sex			
M	761	93 (12.2)	0.60
F	1,011	116 (11.5)	
Age, y			
5–14	396	41 (8.1)	0.35
15–39	921	104 (11.9)	
≥40	455	63 (14.9)	
Skin color			
Nonwhite	1,662	199 (12.0)	0.39
White	110	10 (9.1)	
Household per capita income in US$/day*			
≤5.50	1,429	171 (12.0)	0.69
>5.50	340	37 (10.9)	
Education			
Illiterate	76	14 (18.4)	0.06
Literate	1,696	195 (11.5)	
Occupation/work			
Yes	604	60 (9.9)	0.08
No	1,164	148 (12.7)	
Residence located in an unpaved street			
Yes	1,003	139 (13.9)	0.02
No	767	70 (9.1)	
Type of residence construction			
Plastered wall	1,447	154 (10.6)	0.04
Unplastered wall	211	33 (15.6)	
Wood or other material	106	21 (19.8)	
Residents per household			
1	145	13 (9.0)	0.31
2–3	676	89 (13.2)	
4–5	608	60 (9.0)	
≥6	340	5 (13.5)	
Clinical: reported symptoms†			
Fever and arthralgia			
None	1,212	111 (9.2)	<0.01
Only fever	322	38 (11.8)	
Only arthralgia	89	20 (22.5)	
Both, not simultaneous	40	7 (17.5)	
Both, simultaneous	96	32 (33.3)	
Myalgia			
Yes	222	42 (18.9)	<0.01
No	1,548	167 (10.8)	
Rash			
Yes	216	50 (23.2)	<0.01
No	1,554	158 (10.2)	
Pruritus			
Yes	206	46 (22.3)	<0.01
No	1,563	163 (10.4)	
Presumptive clinical diagnosis			
Chikungunya			
Yes	48	24 (50.0)	<0.01
No	1,724	185 (10.7)	
Dengue			
Yes	111	21 (18.9)	0.02
No	1,661	188 (11.3)	
Zika			
Yes	147	38 (25.9)	<0.01
No	1,625	171 (10.5)	

*Data not shown for 3 participants. †Reported symptoms with onset after January 2015.

The only sociodemographic characteristic associated with previous CHIKV infection in the multiple variable analyses was residence on an unpaved street (prevalence ratio [PR] 1.52, 95% CI 1.07–2.15) (Table 2). In addition, independent clinical predictors for previous CHIKV infection included recall of a presumptive medical diagnosis of chikungunya (PR 2.83, 95% CI 1.97–4.05) and report of an episode of fever with arthralgia (PR 2.26, 95% CI 1.43–3.57) after January 2015, but not for separate episodes of fever or arthralgia (Table 2). 

**Table 2:** Crude and adjusted prevalence ratios for persons with previous chikungunya virus infection, by demographic and clinical characteristics, Salvador, Brazil, November 2016–February 2017

Characteristic	Crude prevalence ratio (95% CI)*	Adjusted prevalence ratio (95% CI)†
Sociodemographic		Model 1
Illiteracy	1.60 (0.99–2.60)	
Not working	1.28 (0.97–1.68)	
Residence located in an unpaved street	1.52 (1.07–2.15)	1.52 (1.07–2.15)
Type of residence construction		
Plastered wall	Referent	
Unplastered wall	1.47 (0.92–2.35)	
Wood/Other material	1.86 (1.06–3.28)	
Clinical: reported symptoms‡		Model 2
Fever and arthralgia		
None	Referent	Referent
Only fever	1.29 (0.89–1.86)	0.96 (0.62–1.49)
Only arthralgia	2.45 (1.60–3.75)	1.55 (0.95–2.53)
Both, not simultaneous	1.91 (0.97–3.77)	1.22 (0.56–2.67)
Both, simultaneous	3.64 (2.51–5.28)	2.26 (1.43–3.57)
Myalgia	1.75 (1.23–2.50)	
Rash	2.28 (1.68–3.08)	
Pruritus	2.14 (1.51–3.03)	
Presumptive clinical diagnosis		
Chikungunya	4.66 (3.35–6.48)	2.83 (1.97–4.05)
Dengue	1.67 (1.09–2.56)	
Zika	2.45 (1.78–3.39)	

*Crude prevalence ratios shown for variables with bivariate p values <0.20, selected for inclusion in the initial multiple variable model. †Two different multiple variable models were applied using backward selection. The first model included only sociodemographic variables to investigate potential exposures associated with CHIKV infection; the second model included only clinical characteristics to investigate predictors of seropositivity.  ‡Reported symptoms with onset after January 2015.

### Frequency of Symptomatic Infections among Participants with CHIKV IgG

Of the 209 participants with detected CHIKV IgG, 32 (15.3%) recalled an episode of fever and arthralgia after January 2015. The median duration of arthralgia for these 32 positive participants was 5 (IQR 3–9) days; the longest duration was 60 days for 1 person. Participants with symptomatic infection tended to be older (p = 0.07); more frequently reported other clinical manifestations compatible with CHIKV infection, such as myalgia, rash, and pruritus (p<0.01 for each symptom); and more commonly received a presumptive clinical diagnosis of chikungunya or Zika (p<0.01 for both) but not of dengue (p = 0.62) (Table 3).

**Table 3:** Comparison of sociodemographic and clinical characteristic of participants with symptomatic versus asymptomatic chikungunya virus infection Salvador, Brazil, November 2016 to February 2017*

Characteristic	Disease status of infected participants, no. %†		p value
	Symptomatic, n = 32	Asymptomatic, n = 177	
Sociodemographic			
Sex			
M	12 (37.5)	81 (45.8)	0.39
F	20 (62.5)	96 (54.2)	
Age, y			
5–14	2 (6.3)	40 (22.6)	0.07
15–39	22 (68.8)	82 (46.3)	
≥40	8 (24.9)	55 (31.1)	
Education‡			
Illiterate	1 (3.1)	13 (7.4)	0.42
Literate	31 (96.9)	163 (92.6)	
Skin color			
White	0	10 (5.7)	NA
Nonwhite	32 (100)	167 (94.3)	
Household per capita income, US$/day‡			
≤5.50	27 (84.4)	144 (81.8)	0.73
>5.50	5 (15.6)	32 (18.2)	
Clinical: reported symptoms			
Fever and arthralgia			
None	0	111 (62.7)	<0.01
Only fever	0	39 (22.0)	
Only arthralgia	0	20 (11.3)	
Both, not simultaneous	0	7 (4.0)	
Both, simultaneous	32 (100)	0	
Myalgia			
Yes	18 (56.3)	24 (13.6)	<0.01
No	14 (43.7)	153 (86.4)	
Rash			
Yes	22 (68.7)	28 (15.9)	<0.01
No	10 (31.3)	148 (84.1)	
Pruritus			
Yes	21 (65.6)	25 (14.1)	<0.01
No	11 (34.4)	152 (85.9)	
Presumptive clinical diagnosis			
Chikungunya			
Yes	20 (62.5)	12 (6.8)	<0.01
No	12 (37.5)	165 (93.2)	
Dengue			
Yes	4 (12.5)	17 (9.6)	0.62
No	28 (87.5)	160 (90.4)	
Zika			
Yes	18 (56.3)	20 (11.3)	<0.01
No	14 (43.7)	157 (88.7%)	

*NA, not available †CHIKV disease status was defined as symptomatic on the basis of self-reported fever accompanied by arthralgia after January 2015. ‡Data not available for 1 participant with an asymptomatic CHIKV infection.

### Frequency of Presumptive Clinical Diagnosis of Chikungunya

Among the 209 participants with a previous CHIKV infection, 24 (11.5%) reported receiving a clinical presumptive diagnosis of chikungunya. Although low, this frequency was 7.5 (95% CI 4.3–12.9) times greater than the 1.5% (24/1,563) frequency among the participants who were negative for CHIKV IgG (p<0.01). Noteworthy for the 32 CHIKV-infected participants who had symptomatic disease, 20 (62.5%) reported a presumptive clinical diagnosis of chikungunya (Table 3). On the other hand, of the 48 participants who reported having received a clinical presumptive diagnosis of chikungunya, 24 had CHIKV IgG detected, indicating a positive predictive value of 50% for the presumptive diagnosis.

### Frequency of CHIKV IgM 

Among the 209 participants who were positive for CHIKV IgG, 49 (23.4%) also had CHIKV IgM, possibly indicating a recent infection. We found no associations between sociodemographic or clinical characteristics and the presence of CHIKV IgM (data not shown).

## Discussion

Despite retrospective evidence of a chikungunya outbreak in Salvador during June–November 2015 (*12*,*15*), we found that ≈2 years later (November 2016–February 2017), <12% of the subjects enrolled in this large cross-sectional neighborhood survey had been infected by CHIKV. This seroprevalence is much lower than that found in 3 additional CHIKV serologic surveys performed in Brazil at that time. During November–December 2015, in Feira de Santana, ≈100 km from Salvador, the prevalence of prior CHIKV infection was estimated at 57.1%; in the urban area of Riachão do Jacuípe, 185 km from Salvador, prevalence was estimated at 45.7% (*29*). In the rural area of Riachão do Jacuípe, the prevalence of prior CHIKV infection was 20.0% in April 2016 (*30*). It is unlikely that a gradual decrease in the IgG levels over time influenced these differences, because we surveyed the participants relatively soon after the outbreak. Thus, the wide range of prevalence levels in adjacent cities most possibly indicates that the intensity of CHIKV transmission, after its first introduction, may vary greatly even among relatively close locations. 

Serum surveys performed in Haiti during December 2014 and February 2015, about 1 year after detection of the index case in the country, also found large variations in the seroprevalence (mean of 78.4% for the urban sites and 44.9% for the rural sites) (*31*). These differences may be related to *Aedes* spp. infestation levels and diversity, variations in local geographic and climate conditions, the predominant CHIKV strain circulating, and even by interactions when the vector species may be coinfected with CHIKV and other circulating arboviruses, such as ZIKV and DENV. Furthermore, a very localized and self-restricted CHIKV outbreak has been recently described in Salvador (*32*), which suggests that local environmental characteristics and patterns of human activity and movement in specific regions may be responsible for the emergence of CHIKV and the extent of its spread. 

In bivariate analyses, we found that structural deficiencies in the housing and on the streets where the houses were located were associated with previous CHIKV infection, pointing to a social gradient that poses an increased risk for virus exposure among the most vulnerable residents. Urban areas served by unpaved streets and where the walls of houses are not plastered, or made of wood or of other material other than brick, often also lack basic sanitation services, such as regular garbage collection and potable water. These conditions, in turn, influence improper disposal of trash and accumulation of water in containers, both well-known breeding grounds for *Aedes* mosquitoes. 

In addition, low education levels in such settings may limit residents’ ability to access, understand, and act on information about measures to prevent mosquitoborne diseases (*33*,*34*). Individual- and ecologic-level studies in rural Kenya (*35*), Nicaragua (*36*), and Colombia (*37*) have also showed that socioeconomic vulnerability and living near sites where water accumulates are associated with increased chikungunya incidence. 

The widely accepted understanding of CHIKV infection has been that the majority (>70%) of infected persons develop a symptomatic form of the disease (*1*). We, conversely, found a frequency of symptomatic infection, defined by having arthralgia accompanied by fever, of only 15.3%. Other studies have also found low proportions of symptomatic CHIKV infection. In Brazil, serologic surveys estimated the proportion of symptomatic CHIKV infection to be 32.7% in Feira de Santana and 41.2% in Riachão do Jacuípe (*29*). Prospective cohort studies, a more robust study design for determining the natural history of disease, have also found low proportions of symptomatic infections. For example, during a cohort follow-up in the Philippines, the subclinical incidence of CHIKV infection was 10.0 per 100 person-years, while the incidence of symptomatic CHIKV infection was 2.2 per 100 person-years, indicating that <20% of those infected exhibited symptoms (*38*). However, because of the small geographic range of the studies, these findings should be considered limited. 

Differences in symptomatic infection rates may be related to the lineage of CHIKV that is circulating (*39*,*40*), the diversity in human immunological responses driven by specific genetic characteristics (*41*), or even by the CHIKV exposure dose delivered by mosquitoes (*42*). In our study, we found that both women and persons ≥15 years of age were more likely to have symptomatic CHIKV infections than others, but the power of our analyses was limited by the small number of CHIKV infections that we detected. However, our results are in accordance with other studies that suggest that women are at increased risk for symptomatic disease and that risk for symptomatic disease increases with age (*29*). Further cohort studies are needed to determine the factors that may influence whether the infection becomes symptomatic. We also found that chronic arthralgia after CHIKV infection was uncommon; the maximum reported duration for the articular pain was 60 days, observed in just 1 (0.5%) of the 209 CHIKV-infected persons. 

On the basis of these findings, we hypothesize that asymptomatic and milder clinical manifestations with less severe arthralgia and low rates of the disease becoming chronic may occur under certain circumstances of CHIKV infection. If further investigation supports this hypothesis, this finding might partially explain the low proportion of participants testing positive for CHIKV who received a correct presumptive diagnosis. 

We did find that report of a presumptive clinical diagnosis of chikungunya disease was strongly associated with having CHIKV IgG (positive predictive value of 50%). Thus, during and after outbreaks, persons exhibiting CHIKV-associated symptoms and suspected disease should be clinically tested because of the likelihood of having confirmed chikungunya disease. 

Our study findings have limitations. First, we surveyed just 1 neighborhood of Salvador and, thus, could not capture potential variations in prior exposure to CHIKV within the city. However, because the community where we conducted the study has poor sanitation infrastructure, which is associated with a higher density of *Aedes aegypti* mosquitoes, and high population density, associated with greater risk of arboviral transmission, it is unlikely that the CHIKV seroprevalence of the city population overall was much higher than the one we measured in the Pau da Lima community. 

Second, we used a commercial CHIKV IgG ELISA to detect previous CHIKV infections. Prior studies have reported high accuracy levels for this test (sensitivity 88%–100%, specificity 82%–95% (*43*,*44*). In our ELISA retesting of 100 IgG negative samples, we found that cryoglobulinemia likely did not influence our seroprevalence; moreover, we found an excellent agreement between the IgG ELISA and the PRNT_90_. However, because cryoglobulinemia had been described for CHIKV (*45*), further surveys should consider this possible effect. 

Third, although transmission of other alphaviruses, such as Mayaro and o’nyong-nyong, has not been reported in northeastern Brazil, because we did not perform PRNT_90_ for other alphaviruses, we cannot completely rule out the possibility of cross-reactions. In addition, it has been shown that IgG seroconversion might not occur or may occur at later stages after CHIKV infection, possibly due to a strong and longlasting CHIKV IgM immune response (*46*)*.* It is possible that this diagnostic limitation hampered detection of some cases of CHIKV infection, especially those occurring shortly before the survey was conducted. 

Fourth, the proportion of symptomatic infections may have been underestimated because of the 2-year gap between the chikungunya outbreak in Salvador and when the study was conducted and because we did not consider those reporting only fever or only arthralgia to have symptomatic disease. Thus, the observed symptomatic rate from our study should be considered a minimum level. Last, the cross-sectional design made it difficult to determine the temporal relation between exposures to risk and occurrence of CHIKV infection. 

In summary, our findings suggest that although CHIKV and ZIKV both spread through Salvador in the same year, 2015 (*12*,*15*,*47*), transmission of CHIKV seems to have been much less intense, reaching ≈12% of the population, compared to estimates of 63%–73% for ZIKV (*22*,*48*). Viral competition within hosts and vectors may be a key element in explaining this dynamic. Further comparative studies on immunopathogenesis and vectorial competence are needed to clarify why these 2 arboviruses, transmitted by the same mosquito vectors, presented such different patterns of transmission spread, given that the population was completely naive for both of them. 

Our findings also show that other parts of Brazil and the Americas may be largely susceptible to CHIKV transmission. It is thus necessary to maintain surveillance to promptly detect further epidemics and to invest in developing and evaluating target interventions, such as vaccines and novel approaches for vector control, that will help protect the population from CHIKV and other arboviral infections. 

Appendix Survey used to collect data for study of transmission of chikungunya virus in an urban slum, Brazil.

## References

[1] Weaver SC, Lecuit M. Chikungunya virus and the global spread of a mosquito-borne disease. N Engl J Med. 2015;372:1231–9. 10.1056/NEJMra1406035 25806915

[2] Thiberville SD, Moyen N, Dupuis-Maguiraga L, Nougairede A, Gould EA, Roques P, et al. Chikungunya fever: epidemiology, clinical syndrome, pathogenesis and therapy. Antiviral Res. 2013;99:345–70. 10.1016/j.antiviral.2013.06.009 23811281 PMC7114207

[3] Fischer M, Staples JE; Arboviral Diseases Branch, National Center for Emerging and Zoonotic Infectious Diseases, CDC. Notes from the field: chikungunya virus spreads in the Americas - Caribbean and South America, 2013-2014. MMWR Morb Mortal Wkly Rep. 2014;63:500–1. 24898168 PMC5779358

[4] Gallian P, Leparc-Goffart I, Richard P, Maire F, Flusin O, Djoudi R, et al. Epidemiology of chikungunya virus outbreaks in Guadeloupe and Martinique, 2014: an observational study in volunteer blood donors. PLoS Negl Trop Dis. 2017;11:e0005254. 10.1371/journal.pntd.0005254 28081120 PMC5230756

[5] Sharp TM, Roth NM, Torres J, Ryff KR, Pérez Rodríguez NM, Mercado C, et al.; Centers for Disease Control and Prevention (CDC). Chikungunya cases identified through passive surveillance and household investigations—Puerto Rico, May 5-August 12, 2014. MMWR Morb Mortal Wkly Rep. 2014;63:1121–8. 10.15585/mmwr.mm6518e3 25474032 PMC4584601

[6] Hennessey MJ, Ellis EM, Delorey MJ, Panella AJ, Kosoy OI, Kirking HL, et al. Seroprevalence and symptomatic attack rate of chikungunya virus infection, United States Virgin Islands, 2014–2015. Am J Trop Med Hyg. 2018;99:1321–6. 10.4269/ajtmh.18-0437 30226143 PMC6221240

[7] Nunes MRT, Faria NR, de Vasconcelos JM, Golding N, Kraemer MU, de Oliveira LF, et al. Emergence and potential for spread of Chikungunya virus in Brazil. BMC Med. 2015;13:102. 10.1186/s12916-015-0348-x 25976325 PMC4433093

[8] Teixeira MG, Andrade AMS, Costa MC, Castro JN, Oliveira FLS, Goes CSB, et al. East/Central/South African genotype chikungunya virus, Brazil, 2014. Emerg Infect Dis. 2015;21:906–7. 10.3201/eid2105.141727 25898939 PMC4412231

[9] Secretaria de Vigilância em Saúde/Ministério da Saúde. Monitoring of cases of dengue, chikungunya fever and fever by the Zika virus until epidemiological week 49, 2016 [in Portuguese]. Vol. 47, Boletim Epidemiológico. 2016 [cited 2018 Dec 23]. https://www.saude.gov.br/images/pdf/2016/dezembro/20/2016-033---Dengue-SE49-publicacao.pdf

[10] Secretaria de Vigilância em Saúde/Ministério da Saúde. Monitoring of cases of dengue, chikungunya fever and fever by the Zika virus until epidemiological week 35, 2017 [In Portuguese]. Vol. 48, Boletim Epidemiológico. 2017 [cited 2018 Dec 23]. https://www.saude.gov.br/images/pdf/2017/setembro/15/2017-028-Monitoramento-dos-casos-de-dengue--febre-de-chikungunya-e-febre-pelo-virus-Zika-ate-a-Semana-Epidemiologica-35.pdf

[11] Secretaria de Vigilância em Saúde/Ministério da Saúde. Monitoring of cases of dengue, chikungunya fever and fever by the Zika virus until epidemiological week 43, 2018 [in Portuguese]. Vol. 49, Boletim Epidemiológico. 2018 [cited 2018 Dec 23]. http://portalarquivos2.saude.gov.br/images/pdf/2018/novembro/13/2018-056.pdf

[12] Cardoso CW, Kikuti M, Prates APPB, Paploski IAD, Tauro LB, Silva MMO, et al. Unrecognized emergence of chikungunya virus during a Zika virus outbreak in Salvador, Brazil. PLoS Negl Trop Dis. 2017;11:e0005334. 10.1371/journal.pntd.0005334 28114414 PMC5289630

[13] de Oliveira WK, Carmo EH, Henriques CM, Coelho G, Vazquez E, Cortez-Escalante J, et al. Zika virus infection and associated neurologic disorders in Brazil. N Engl J Med. 2017;376:1591–3. 10.1056/NEJMc1608612 28402236 PMC5544116

[14] Instituto Brasileiro de Geografia e Estatística. Brazil/Bahia/Salvador. 2018 [cited 2019 Jun 5]. https://cidades.ibge.gov.br/brasil/ba/salvador/panorama

[15] Silva MMO, Tauro LB, Kikuti M, Anjos RO, Santos VC, Gonçalves TSF, et al. Concomitant transmission of dengue, chikungunya and Zika viruses in Brazil: clinical and epidemiological findings from surveillance for acute febrile illness. Clin Infect Dis. 2019;69:1353–9. 10.1093/cid/ciy1083 30561554 PMC7348233

[16] Kikuti M, Cunha GM, Paploski IAD, Kasper AM, Silva MM, Tavares AS, et al. Spatial distribution of dengue in a Brazilian urban slum setting: role of socioeconomic gradient in disease risk. PLoS Negl Trop Dis. 2015;9:e0003937. 10.1371/journal.pntd.0003937 26196686 PMC4510880

[17] Reis RB, Ribeiro GS, Felzemburgh RDM, Santana FS, Mohr S, Melendez AXTO, et al. Impact of environment and social gradient on *Leptospira* infection in urban slums. PLoS Negl Trop Dis. 2008;2:e228. 10.1371/journal.pntd.0000228 18431445 PMC2292260

[18] Felzemburgh RDM, Ribeiro GS, Costa F, Reis RB, Hagan JE, Melendez AXTO, et al. Prospective study of leptospirosis transmission in an urban slum community: role of poor environment in repeated exposures to the *Leptospira* agent. PLoS Negl Trop Dis. 2014;8:e2927. 10.1371/journal.pntd.0002927 24875389 PMC4038618

[19] Hagan JE, Moraga P, Costa F, Capian N, Ribeiro GS, Wunder EA Jr, et al. Spatiotemporal determinants of urban leptospirosis transmission: four-year prospective cohort study of slum residents in Brazil. PLoS Negl Trop Dis. 2016;10:e0004275. 10.1371/journal.pntd.0004275 26771379 PMC4714915

[20] Rodriguez-Barraquer I, Costa F, Nascimento EJM, Nery N, Castanha PMS, Sacramento GA, et al. Impact of preexisting dengue immunity on Zika virus emergence in a dengue endemic region. Science. 2019;363:607–10. 10.1126/science.aav6618 30733412 PMC8221194

[21] Silva MM, Rodrigues MS, Paploski IA, Kikuti M, Kasper AM, Cruz JS, et al. Accuracy of dengue reporting by national surveillance system, Brazil. Emerg Infect Dis. 2016;22:336–9. 10.3201/eid2202.150495 26812472 PMC4734515

[22] Unger A, Felzemburgh RDM, Snyder RE, Ribeiro GS, Mohr S, Costa VBA, et al.; Pau da Lima Urban Health Team. Hypertension in a Brazilian urban slum population. J Urban Health. 2015;92:446–59. 10.1007/s11524-015-9956-1 25920334 PMC4456479

[23] Snyder RE, Rajan JV, Costa F, Lima HCAV, Calcagno JI, Couto RD, et al. Differences in the prevalence of non-communicable disease between slum dwellers and the general population in a large urban area in Brazil. Trop Med Infect Dis. 2017;2:47. 10.3390/tropicalmed2030047 30270904 PMC6082112

[24] Harvey LA. REDCap: web-based software for all types of data storage and collection. Spinal Cord. 2018;56:625. 10.1038/s41393-018-0169-9 29977003

[25] Baer A, Kehn-Hall K. Viral concentration determination through plaque assays: using traditional and novel overlay systems. J Vis Exp. 2014; (93):e52065. 10.3791/52065 25407402 PMC4255882

[26] Kolopp-Sarda MN, Miossec P. Cryoglobulins: An update on detection, mechanisms and clinical contribution. Autoimmun Rev. 2018;17:457–64. 10.1016/j.autrev.2017.11.035 29526627

[27] World Bank. Piecing together the poverty puzzle. 2018 [cited 2018 Dec 21]. https://openknowledge.worldbank.org/bitstream/handle/10986/30418/9781464813306.pdf

[28] StataCorp. Stata Statistical Software: release 14. College Station (TX): StataCorp LP. 2015 [cited 2018 Sep 30]. https://www.stata.com

[29] Dias JP, Costa MDCN, Campos GS, Paixão ES, Natividade MS, Barreto FR, et al. Seroprevalence of chikungunya virus after its emergence in Brazil. Emerg Infect Dis. 2018;24:617–24. 10.3201/eid2404.171370 29553317 PMC5875253

[30] Cunha RV, Trinta KS, Montalbano CA, Sucupira MVF, de Lima MM, Marques E, et al. Seroprevalence of chikungunya virus in a rural community in Brazil. PLoS Negl Trop Dis. 2017;11:e0005319. 10.1371/journal.pntd.0005319 28107342 PMC5287455

[31] Rogier EW, Moss DM, Mace KE, Chang M, Jean SE, Bullard SM, et al. Use of bead-based serologic assay to evaluate chikungunya virus epidemic, Haiti. Emerg Infect Dis. 2018;24:995–1001. 10.3201/eid2406.171447 29774861 PMC6004842

[32] Tauro LB, Cardoso CW, Souza RL, Nascimento LC, Santos DRD, Campos GS, et al. A localized outbreak of Chikungunya virus in Salvador, Bahia, Brazil. Mem Inst Oswaldo Cruz. 2019;114:e180597. 10.1590/0074-02760180597 30843962 PMC6396974

[33] Higuera-Mendieta DR, Cortés-Corrales S, Quintero J, González-Uribe C. KAP surveys and dengue control in Colombia: disentangling the effect of sociodemographic factors using multiple correspondence analysis. PLoS Negl Trop Dis. 2016;10:e0005016. 10.1371/journal.pntd.0005016 27682141 PMC5040257

[34] Whiteman A, Mejia A, Hernandez I, Loaiza JR. Socioeconomic and demographic predictors of resident knowledge, attitude, and practice regarding arthropod-borne viruses in Panama. BMC Public Health. 2018;18:1261. 10.1186/s12889-018-6172-4 30428861 PMC6236898

[35] Grossi-Soyster EN, Cook EAJ, de Glanville WA, Thomas LF, Krystosik AR, Lee J, et al. Serological and spatial analysis of alphavirus and flavivirus prevalence and risk factors in a rural community in western Kenya. PLoS Negl Trop Dis. 2017;11:e0005998. 10.1371/journal.pntd.0005998 29040262 PMC5659799

[36] Kuan G, Ramirez S, Gresh L, Ojeda S, Melendez M, Sanchez N, et al. Seroprevalence of anti-chikungunya virus antibodies in children and adults in Managua, Nicaragua, after the first chikungunya epidemic, 2014–2015. PLoS Negl Trop Dis. 2016;10:e0004773. 10.1371/journal.pntd.0004773 27322692 PMC4913910

[37] Krystosik AR, Curtis A, Buritica P, Ajayakumar J, Squires R, Dávalos D, et al. Community context and sub-neighborhood scale detail to explain dengue, chikungunya and Zika patterns in Cali, Colombia. PLoS One. 2017;12:e0181208. 10.1371/journal.pone.0181208 28767730 PMC5540594

[38] Yoon I-K, Alera MT, Lago CB, Tac-An IA, Villa D, Fernandez S, et al. High rate of subclinical chikungunya virus infection and association of neutralizing antibody with protection in a prospective cohort in the Philippines. PLoS Negl Trop Dis. 2015;9:e0003764. 10.1371/journal.pntd.0003764 25951202 PMC4423927

[39] Teo T-H, Her Z, Tan JJL, Lum F-M, Lee WWL, Chan Y-H, et al. Caribbean and La Réunion chikungunya virus isolates differ in their capacity to induce proinflammatory Th1 and NK cell responses and acute joint pathology. J Virol. 2015;89:7955–69. 10.1128/JVI.00909-15 25995257 PMC4505608

[40] Langsjoen RM, Haller SL, Roy CJ, Vinet-Oliphant H, Bergren NA, Erasmus JH, et al. Chikungunya virus strains show lineage-specific variations in virulence and cross-protective ability in murine and nonhuman primate models. MBio. 2018;9:e02449–17. 10.1128/mBio.02449-17 29511072 PMC5844994

[41] Chaaithanya IK, Muruganandam N, Anwesh M, Rajesh R, Ghosal SR, Kartick C, et al. HLA class II allele polymorphism in an outbreak of chikungunya fever in Middle Andaman, India. Immunology. 2013;140:202–10. 10.1111/imm.12128 23710940 PMC3784166

[42] Gordon A, Gresh L, Ojeda S, Chowell G, Gonzalez K, Sanchez N, et al. Differences in transmission and disease severity between 2 successive waves of chikungunya. Clin Infect Dis. 2018;67:1760–7. 10.1093/cid/ciy356 29697796 PMC6233685

[43] Prat CM, Flusin O, Panella A, Tenebray B, Lanciotti R, Leparc-Goffart I. Evaluation of commercially available serologic diagnostic tests for chikungunya virus. Emerg Infect Dis. 2014;20:2129–32. 10.3201/eid2012.141269 25418184 PMC4257799

[44] De Salazar PM, Valadere AM, Goodman CH, Johnson BW. Evaluation of three commercially-available chikungunya virus immunoglobulin G immunoassays. Rev Panam Salud Publica. 2017;41:e62. 10.26633/RPSP.2017.62 28902275 PMC6612722

[45] Oliver M, Grandadam M, Marimoutou C, Rogier C, Botelho-Nevers E, Tolou H, et al. Persisting mixed cryoglobulinemia in Chikungunya infection. PLoS Negl Trop Dis. 2009;3:e374. 10.1371/journal.pntd.0000374 19190731 PMC2629124

[46] Bozza FA, Moreira-Soto A, Rockstroh A, Fischer C, Nascimento AD, Calheiros AS, et al. Differential shedding and antibody kinetics of Zika and chikungunya viruses, Brazil. Emerg Infect Dis. 2019;25:311–5. 10.3201/eid2502.180166 30666934 PMC6346451

[47] Cardoso CW, Paploski IAD, Kikuti M, Rodrigues MS, Silva MMO, Campos GS, et al. Outbreak of exanthematous illness associated with Zika, chikungunya, and dengue viruses, Salvador, Brazil. Emerg Infect Dis. 2015;21:2274–6. 10.3201/eid2112.151167 26584464 PMC4672408

[48] Netto EM, Moreira-Soto A, Pedroso C, Höser C, Funk S, Kucharski AJ, et al. High Zika virus seroprevalence in Salvador, Northeastern Brazil limits the potential for further outbreaks. MBio. 2017;8:e01390–407. 10.1128/mBio.01390-17 29138300 PMC5686533

