# Supplementary material for: Transmission of Chikungunya Virus in an Urban Slum, Brazil
Source: Emerg Infect Dis. 2020 Jul;26(7):1364–73. doi: 10.3201/eid2607.190846 (PMC7323528; doi:10.3201/eid2607.190846)
Supplement: Appendix — Survey used to collect data for study of transmission of chikungunya virus in an urban slum, Brazil. [file 19-0846-Techapp-s1.pdf]

Article DOI: https://doi.org/10.3201/eid2607.190846

Transmission of Chikungunya Virus in an Urban Slum Setting, Brazil

Appendix

Study Questionnaire

Household Data

House Identification Number ______________________________

Interviewer Initials ______________________________

Date of the interview ______________________________

(Day-Month-Year)

Location of the house

☐ Valley 1 ☐ Valley 2 ☐ Valley 4

House block ______________________________

(01–50)

House number on block ______________________________

(010 - 990)

Home Use

☐ Empty
☐ Abandoned
☐ Construction
☐ Commercial
☐ Residential
☐ Commercial e residential

Household inclusion

☐ Accepted
☐ Refused
☐ Not found
☐ Access impossible

Number of residents in the house ______________________________

Is the access street to your house paved?

☐ Yes
☐ No

What is the predominant material of the walls of your home?

☐ Plastered wall
☐ Unplastered wall
☐ Wood or other material that is not brickwork

Individual Consent Data

Participant identification number ___________________________________

Name? ___________________________________

Sex

☐ Male
☐ Female

Date of birth? ___________________________________
(Day-Month-Year)

How old are you? ___________________________________

Do you accept participate of study?

☐ Yes
☐ No

Date of consent or refusal ___________________________________
(Day-Month-Year)

What is the reason for not giving consent to participate?

☐ Not found
☐ Refused
☐ Mentally incapable
☐ Other

Sociodemographic Data

What is your marital status?

☐ Single
☐ Married
☐ Stable union (>6 months)
☐ Widower
☐ Divorced

What is your skin color?

☐ White
☐ Black
☐ Brown
☐ Other

What was the last grade that you completed?

- ☐ Never studied or never completed the first year of fundamental school
- ☐ 1st year of fundamental school
- ☐ 2nd year of fundamental school
- ☐ 3rd year of fundamental school
- ☐ 4th year of fundamental school
- ☐ 5th year of fundamental school
- ☐ 6th year of fundamental school
- ☐ 7th year of fundamental school
- ☐ 8th year of fundamental school
- ☐ 9th year of fundamental school
- ☐ 1st year of high school
- ☐ 2nd year of high school
- ☐ 3rd year of high school
- ☐ Incomplete college level
- ☐ Complete college level

Are you currently working or doing any work activity (formal or informal) for whom you are paid (Can be formal or informal work)?

- ☐ Yes
- ☐ No

How much do you receive per month? ____________________________________

History of Signs and Symptoms Data

NOTE: The following questions regarding fever and arthralgia were used to identify participants with a history compatible with symptomatic CHIKV infection:

From January 2015 until today did you have a fever?

- ☐ Yes
- ☐ No

If yes, How many times? _____________________

If yes, What was the month and year of the fever (MM/YY)?___________

From January 2015 until today did you have joint pain?

- ☐ Yes
- ☐ No

If yes, How many times?______________________

If yes, What was the month and year of the joint pain (MM/YY)?_______

During the analyses, we identified individuals reporting only fever; only arthralgia; both, not simultaneous (when they occurred in different periods); and both, simultaneous (when they occurred in the same month and year). We considered the CHIKV IgG-positive participants who simultaneously reported both fever and arthralgia after January 2015 as those with symptomatic CHIKV infection.

From January 2015 until today did you have a rash?

☐ Yes
☐ No

If yes, How many times? ____________________

If yes, What was the month and year of the rash (MM/YY)?___________

From January 2015 until today did you have a myalgia?

☐ Yes
☐ No

If yes, How many times? ____________________

If yes, What was the month and year of the myalgia (MM/YY)?________

From January 2015 until today did you have a pruritus?

☐ Yes
☐ No

If yes, How many times? ____________________

If yes, What was the month and year of the pruritus (MM/YY)?______________

Did a doctor ever tell you that you had any of the following diseases (you can select more than one option)?

☐ Leptospirosis
☐ Dengue
☐ Zika
☐ Chikungunya
☐ High pressure
☐ Diabetes
☐ Cancer
☐ Heart disease
☐ Stroke
☐ None
